# Nonanaplastic follicular cell-derived thyroid carcinoma: mitosis and necrosis in long-term follow-up

**DOI:** 10.1007/s00405-017-4527-6

**Published:** 2017-03-14

**Authors:** Daniel Bräuner Skansing, Stefano Christian Londero, Pia Asschenfeldt, Stine Rosenkilde Larsen, Christian Godballe

**Affiliations:** 1 Department of ORL Head & Neck Surgery, Odense University Hospital, Afd. F, Odense Universitetshospital, Sdr. Boulevard 29, 5000 Odense C, Denmark; 2 Department of Pathology, Aalborg University Hospital, Ålborg, Denmark; 3 Department of Pathology, Odense University Hospital, Odense C, Denmark

**Keywords:** Thyroid carcinoma, Long-term survival, Prognostic factors, Tumor necrosis, Mitosis

## Abstract

Nonanaplastic follicular cell-derived thyroid carcinoma (NAFCTC) includes differentiated- (DTC) and poorly differentiated thyroid carcinoma (PDTC). DTC has an excellent prognosis, while PDTC is situated between DTC and anaplastic carcinomas. Short-term studies suggest that PDTC patients diagnosed only on tumor necrosis and/or mitosis have a prognosis similar to those diagnosed according to the TURIN proposal. The purpose of this study was to evaluate prognosis for NAFCTC based on long-term follow-up illuminating the significance of tumor necrosis and mitosis. A cohort of 225 patients with NAFCTC was followed more than 20 years. Age, sex, distant metastasis, histology, tumor size, extrathyroidal invasion, lymph node metastasis, tumor necrosis and mitosis were examined as possible prognostic factors. Median follow-up time for patients alive was 28 years (range 20–43 years). Age, distant metastasis, extrathyroidal invasion, tumor size, tumor necrosis and mitosis were independent prognostic factors in multivariate analysis for overall survival (OS). In disease specific survival (DSS) age was not significant. Using only necrosis and/or mitosis as criteria for PDTC the 5-, 10- and 20-year OS for DTC was 87, 79 and 69%, respectively. In DSS it was 95, 92 and 90%. For PDTC the 5-, 10- and 20-year OS was 57, 40 and 25%, respectively. In DSS it was 71, 55 and 48%. Tumor necrosis and mitosis are highly significant prognostic indicators in analysis of long time survival of nonanaplastic follicular cell-derived thyroid carcinoma indicating that a simplification of the actually used criteria for poorly differentiated carcinomas may be justified.

## Introduction

Thyroid carcinoma is a relatively uncommon cancer accounting for approximately 1% of malignant disease; still it is the most frequent endocrine malignancy [1, 2]. Over the last decades a rise in incidence has been detected in several countries including Denmark and it seems that especially the incidentally found papillary micro-carcinomas are responsible for the increase [3, 4].

Nonanaplastic follicular cell-derived thyroid carcinoma (NAFCTC) includes differentiated- (DTC) and poorly differentiated thyroid carcinoma (PDTC). DTC has an excellent prognosis with a 10-year survival above 90% [5], while PDTC is situated between DTC and anaplastic carcinomas. In 2004, WHO recognized PDTC as a separate histological entity and defined it as “a follicular-cell neoplasm that shows limited evidence of structural follicular cell differentiation and occupy both morphologically and behaviorally an intermediate position between differentiated (follicular and papillary carcinomas) and undifferentiated (anaplastic) carcinoma” [6]. In 2006, Hiltzik et al. found that PDTC defined on the basis of only mitosis and necrosis constitutes a group of tumors that is more aggressive and homogeneous than PDTC defined by the WHO criteria [7]. In 2007, the TURIN proposal was published including necrosis and mitosis, as proposed by Hiltzik et al. but also, it was extended with variables concerning growth pattern (solid, trabecular or insular) and nuclear morphology [8] making interpretation more complicated. Controversy still exists regarding the diagnostic criteria [7, 9, 10]. In 2014, Gnemmi et al. published a comparison of the TURIN proposal with Hiltzik’s histological grade not including growth pattern. They found similar performances in predicting prognosis [11], indicating that the less complicated model from Hiltzik et al. could be a sufficient tool for identification of PDTC. However, the median follow-up time for the Hiltzik and the Gnemmi studies were only 3.5 and 5.7 years, respectively.

The purpose of this study was to evaluate prognosis for NAFCTC based on long-term follow-up with focus on the significance of tumor necrosis and mitosis.

## Materials and methods

From 1970 to 1992, 297 patients with histologically verified primary thyroid carcinoma were treated and/or followed at Odense University Hospital, Denmark—a tertiary head and neck cancer center. Due to the unique Danish personal identity number it was possible to establish a retrospective cohort study of NAFCTC patients with a long-term follow-up. All patients came from a well-defined geographic area, the Region of Southern Denmark. At the end of the inclusion period the same pathologist (PA) did a complete histological revision. Autopsy findings were not included.

Two hundred thirty-one patients had histologically verified NAFCTC. In six cases treatment with curative intent was not possible and they were excluded from further analysis. Two hundred twenty-five patients were eligible for analysis. Three patients were lost to follow-up because of emigration. They were censored according to last available information.

Follow-up procedure for thyroid cancer was performed with a 3-month interval 2 years after treatment and a 6-month interval the following 3 years. In the case of recurrence follow-up procedure would start over. Standard ENT examination was performed. Ultrasound was not yet standard procedure and was not done routinely.

Initial data concerning diagnostic procedures, treatment, and histology has been shown in an earlier publication [12], while new data concerning recurrence, last contact to hospital and cause of death were retrieved as part of the present study. The information was obtained from review of patient charts, contact to general practitioners and death certificates. In survival analysis primary endpoints were death and recurrence. Survival analysis was investigated as overall survival (OS) and disease specific survival (DSS). In OS death from all causes was considered an event. In DSS death from disease or death with disease were considered events. Recurrence was defined as persistent disease or occurrence of disease after the end of primary treatment confirmed by histology, cytology and/or imaging. Patients with a residual tumor after primary treatment were registered with the date of recurrence set to be the date of diagnosis. In a few cases only the month of recurrence was available, thus the date of recurrence was listed as the first of that month. Closing date for the follow-up was March 1, 2013.

TNM staging was performed according to the fourth edition of UICC TNM classification [13]. An update to the seventh edition [14] was not possible due to insufficient information concerning lymph node metastasis and extrathyroidal invasion. Indications for radioactive iodine treatment (RAI) were presence of distant metastasis, lymph node metastasis, extrathyroidal extension and/or non-radical surgery.

Age at the time of diagnosis, sex, distant metastasis, histological type (papillary versus follicular), tumor size, extrathyroidal invasion (pT4), regional lymph node metastasis, tumor necrosis and mitosis and were evaluated as possible prognostic factors. All variables were dichotomized. Age had 45 years as cut-point. For tumor size the median was used as cut-point. Distant metastasis, extrathyroidal invasion, tumor necrosis and regional lymph node metastasis were registered as “present” or “not present”. The presence of mitosis was registered as positive if more than one mitosis per high power field was observed.

Survival curves were generated using the Kaplan–Meier method. In univariate analysis the Mantel-Haenzel log-rank test was used to evaluate differences between groups. *P* values < 0.05 were considered significant. Variables identified as significant for survival in univariate analysis were included in a multivariate Weibull regression model, model fit was checked using Cox-Schnell residuals. Backward selection procedures were performed.

Medlog^®^ (Information Analysis Corporation, Crystal Bay, Nevada 89402 USA) for Windows^®^ version 2012-8 was used for clinical data management and data registration, and SPSS^®^ for MAC version 21 (IBM Corporation, Armonk, New York 10504-1722 USA) and STATA/IC 11 (StataCorp LP, College Station, Texas 77845 USA) were used for statistical analysis.

## Results

### Follow-up

Two hundred twenty-five patients were included in the analysis and the female:male ratio was 2.8:1. The median age at the time of diagnosis was 51 years (range 8–91 years).

The median follow-up time for patients alive was 28 years (range 20–43 years), when the three patients lost to follow-up were not included. They were censored after 3, 4 and 19 years, respectively. One hundred seventeen patients died during the period of follow-up (0.2–34 years after diagnosis). The median age at the time of death was 75 years (range 31–99 years). During follow-up, 117 patients died (thyroid carcinoma 45%; other disease than cancer 37%; other cancer 13%; accidents 2%; complications to treatment 1%; unknown 3%). Sixty-seven patients had recurrence. Stage and treatment of the study group are shown in Table 1.

**Table 1:** TNM stage and treatment for 225 patients with nonanaplastic follicular cell-derived thyroid carcinoma

Characteristics	Patients no.	Percent
TNM stage#		
Stage I	99	44
Stage II	52	23.1
Stage III	44	19.6
Stage IV	30	13.3
Treatment		
Surgical		
T-position		
Open biopsy	2	0.9
Tumorectomy	5	2.2
Hemithyroidectomy	54	24
Subtotal thyroidectomy	51	22.7
Total thyroidectomy	113	50.2
*N*-position		
Lymph node extirpation	49	21.8
Modified neck dissection	48	21.3
Radical neck dissection	25	11.1
Adjuvant		
RAI	96	42.7
External radiotherapy	16	7.1

*RAI* radioactive iodine treatment

#According to International Union Against Cancer (UICC) 1987, 4. edition

### Prognostic factors

Univariate analysis of patient and tumor related prognostic factors are shown in Table 2. In six patients no primary tumor was found leaving 219 patients for analysis of histological features.

**Table 2:** Univariate survival analysis of overall survival and disease specific survival in 225 patients with nonanaplastic follicular cell-derived thyroid carcinoma

Variables	Patients no.	10-year OS	20-year OS	*P* value	10-year DSS	20-year DSS	*P* value
Age							
0–45	95	0.936 (0.9;1.0)	0.912 (0.9;1,0)		0.968 (0.9;1.0)	0.968 (0.9;1.0)	
>45	130	0.431 (0.3;0.5)	0.274 (0.2;0.4)	<0.0001	0.622 (0.5;0.7)	0.560 (0.5;0.7)	<0.0001
Sex							
Female	165	0.641 (0.6;0.7)	0.557 (0.5;0.6)		0.791 (0.7;0.9)	0.774 (0.7;0.8)	
Male	60	0.645 (0.5;0.8)	0.451 (0.3;0.6)	0.4456	0.765 (0.7;0.9)	0.679 (0.6;0.8)	0.2351
Tumor size							
≤35	118	0.821 (0.8;0.9)	0.747 (0.7;0.8)		0.947 (0.9;1.0)	0.947 (0.9;1.0)	
>35	105	0.436 (0.3;0.5)	0.293 (0.2;0.4)	<0.0001	0.572 (0.5;0.7)	0.501 (0.4;0.6)	<0.0001
Lymph node metastasis							
Present	96	0.619 (0.5;0.7)	0.487 (0.4;0.6)		0.722 (0.6;0.8)	0.709 (0.6;0.8)	
Not present	129	0.659 (0.6;0.7)	0.561 (0.5;0.6)	0.5831	0.822 (0.8;0.9)	0.780 (0.7;0.9)	0.1847
Distant metastasis							
Present	36	0.167 (0.1;0.3)	0.111 (0.0;0.2)		0.232 (0.1;0.4)	0.193 (0.1;0.3)	
Not present	189	0.733 (0.7;0.8)	0.618 (0.6;0.7)	<0.0001	0.879 (0.8;0.9)	0.850 (0.8;0.9)	<0.0001
Histology							
Papillary	170	0.722 (0.7;0.8)	0,619 (0.5;0.7)		0.855 (0.8;0.9)	0.833 (0.8;0.9)	
Follicular	55	0.400 (0.3;0.5)	0.272 (0.2;0.4)	<0.0001	0.535 (0.4;0.7)	0.480 (0.3;0.6)	< 0.0001
Extrathyroidal invasion							
Present	73	0.420 (0.3;0.5)	0.317 (0.2;0.4)		0.550 (0.4;0.7)	0.472 (0.3;0.6)	
Not present	145	0.758 (0.7;0.8)	0.646 (0.6;0.7)	<0.0001	0.889 (0.8;0.9)	0.869 (0.8;0.9)	<0.0001
Necrosis							
Present	48	0.208 (0.1;0.3)	0.143 (0.0;0.2)		0.350 (0.2;0.5)	0.280 (0.1;0.4)	
Not present	171	0.770 (0.7;0.8)	0.649 (0.6;0.7)	<0.0001	0.899 (0.9;1.0)	0.870 (0.8;0.9)	<0.0001
Mitosis							
Present	37	0.324 (0.2;0.5)	0.162 (0.0;0.3)		0.418 (0.3;0.6)	0.383 (0.2;0.6)	
Not present	182	0.712 (0.7;0.8)	0.617 (0.6;0.7)	<0.0001	0.857 (0.8;0.9)	0.821 (0.8;0.9)	<0.0001

A total of 218 patients had sufficient tissue for examination of all parameters and were included in a multivariate analysis using death as primary endpoint. The results are shown in Table 3.

**Table 3:** Cox regression analysis of overall survival and disease specific survival in 218 patients with nonanaplastic follicular cell-derived thyroid carcinoma

Variables	OS (111 events)			DSS (52 events)		
	HR	95% CI	*P* value	HR	95% CI	*P* value
Age >45 years	1.8	1.2–2.7	0.004	1.7	0.9–3.0	0.099
Distant metastasis	2.3	1.4–3.6	0.001	3.4	1.8–6.3	< 0.0001
Extrathyroidal invasion	1.7	1.1–2.6	0.010	2.8	1.5–5.2	0.002
Histology	1.3	0.8–2.1	0.232	1.6	0.8–3.0	0.185
Mitosis	2.0	1.3–3.2	0.004	2.0	1.1–3.9	0.032
Necrosis	2.8	1.8–4.4	<0.0001	3.8	2.1–7.0	< 0.0001
Tumor size >35 mm	1.9	1.2–3.0	0.004	3.5	1.4–8.3	0.006

*HR* hazard ratio, *CI* confidence interval

Surgical treatment was dichotomized with total and subtotal thyroidectomies in one group and less extensive procedures in another group. In univariate analysis surgical treatment was not significant. Patients who received RAI had a significantly worse prognosis compared to non-receivers, but this difference disappeared when adjustment for stage was performed.

### Survival

**Fig. 1:**
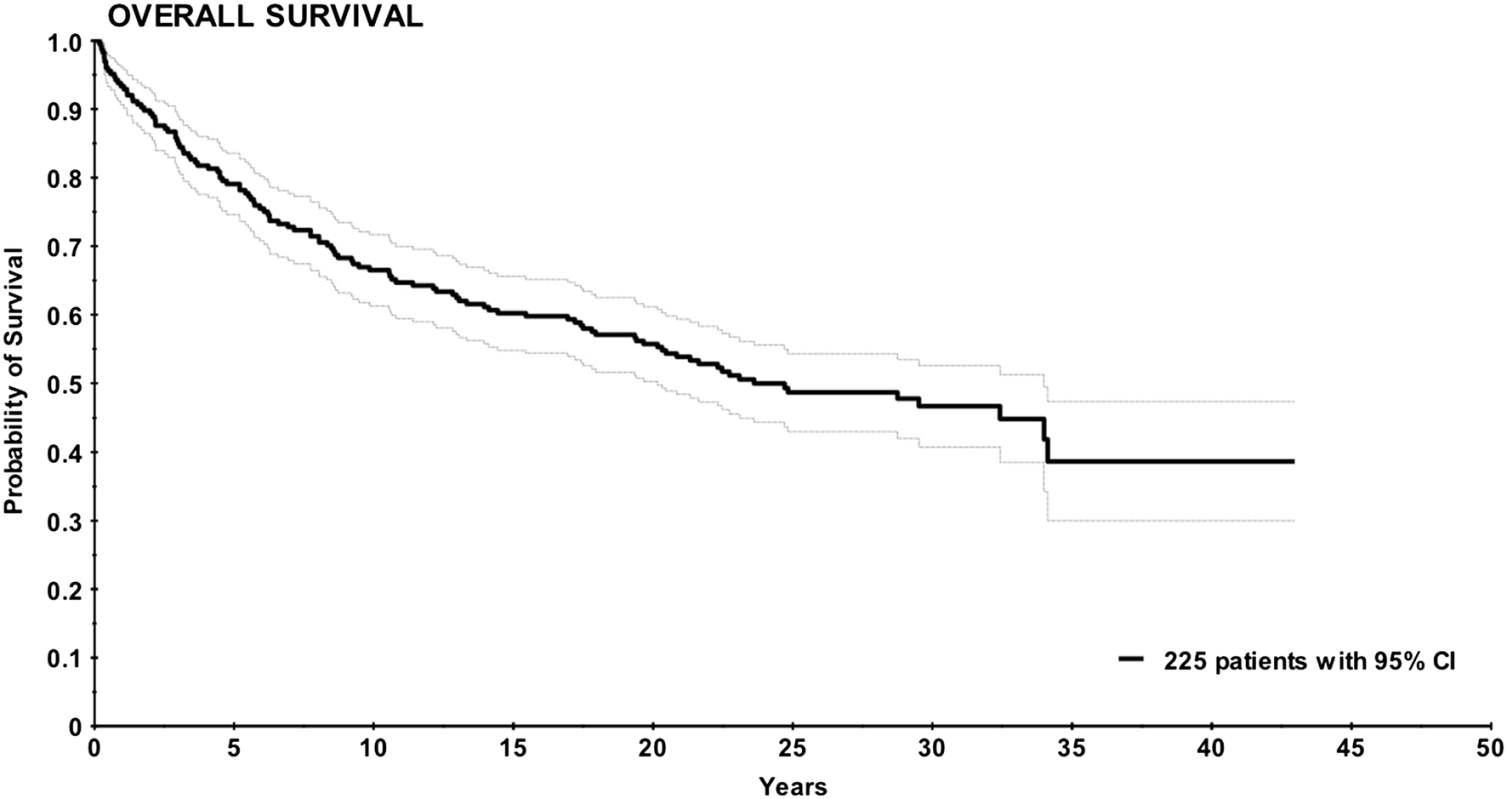
Kaplan–Meier curve for overall survival in 225 patients displayed with 95% confidence interval

**Fig. 2:**
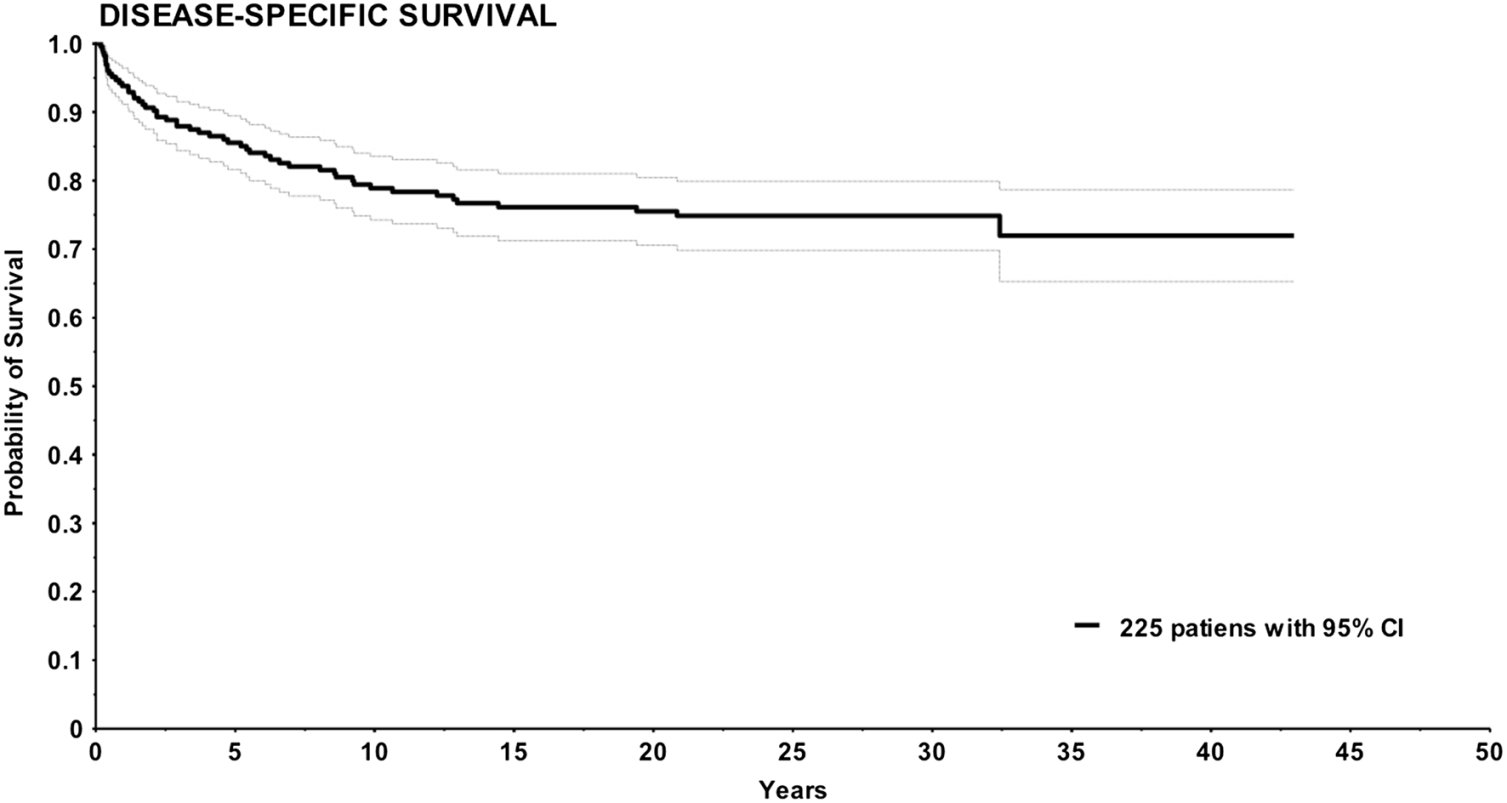
Kaplan–Meier curve for disease specific survival in 225 patients displayed with 95% confidence interval

Figures 1 and 2 show Kaplan–Meier survival curves for NAFCTC. In OS the 5-, 10- and 20-year survival was 75, 65 and 54%, respectively. In DSS 53 the 5-, 10- and 20-year survival was 84, 78 and 75%, respectively.

**Fig. 3:**
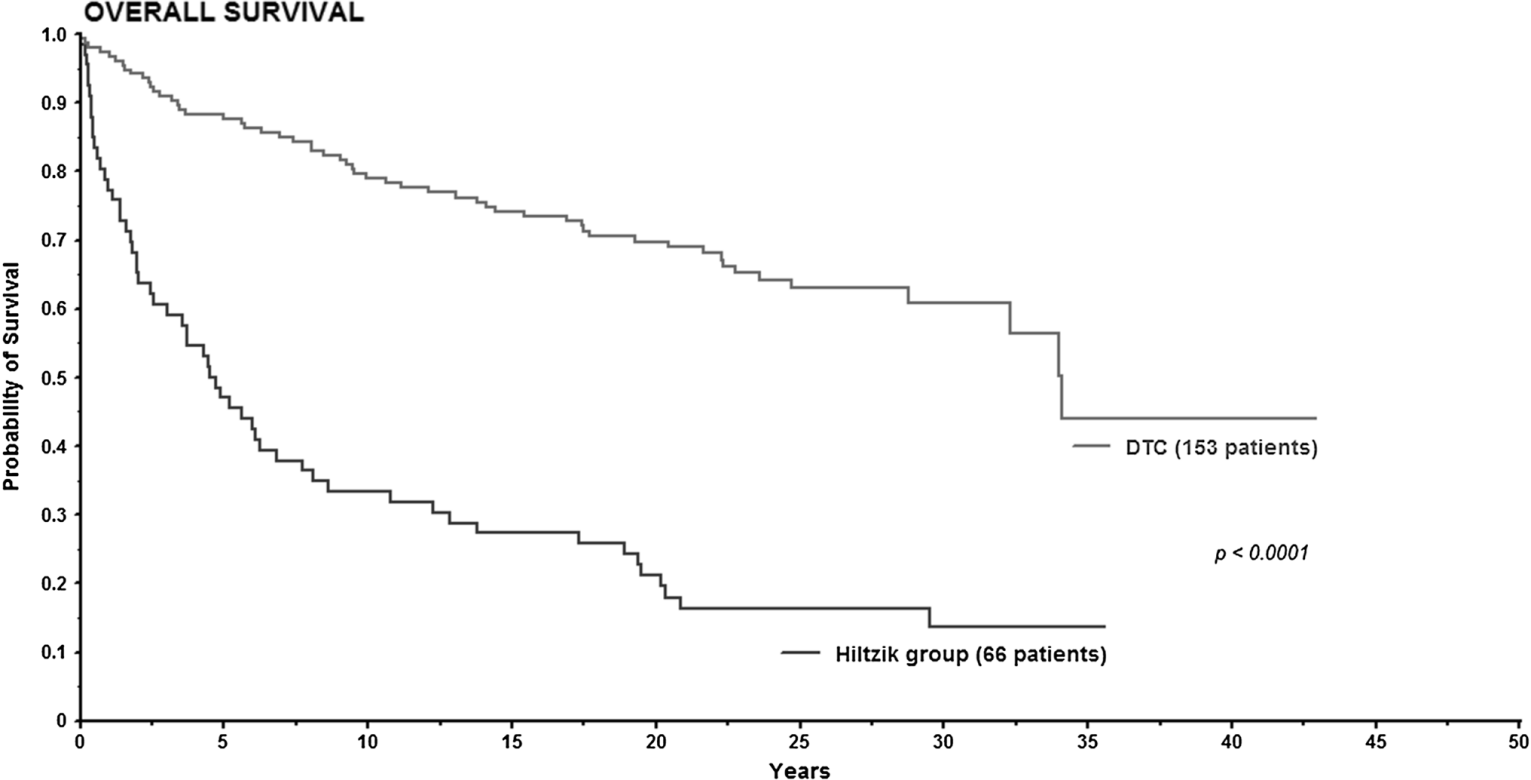
Kaplan–Meier curve for overall survival in differentiated thyroid carcinoma and Hiltzik criteria group

Figure 3 shows Kaplan–Meier survival curves for DTC and Hiltzik criteria group for OS. In the DTC group the 5-, 10- and 20-year survival was 87, 79 and 69%, respectively. In the Hiltzik criteria group the 5-, 10- and 20-year survival was 57, 40 and 25%, respectively. Log rank test showed significantly difference between the groups (*P* < 0.0001).

**Fig. 4:**
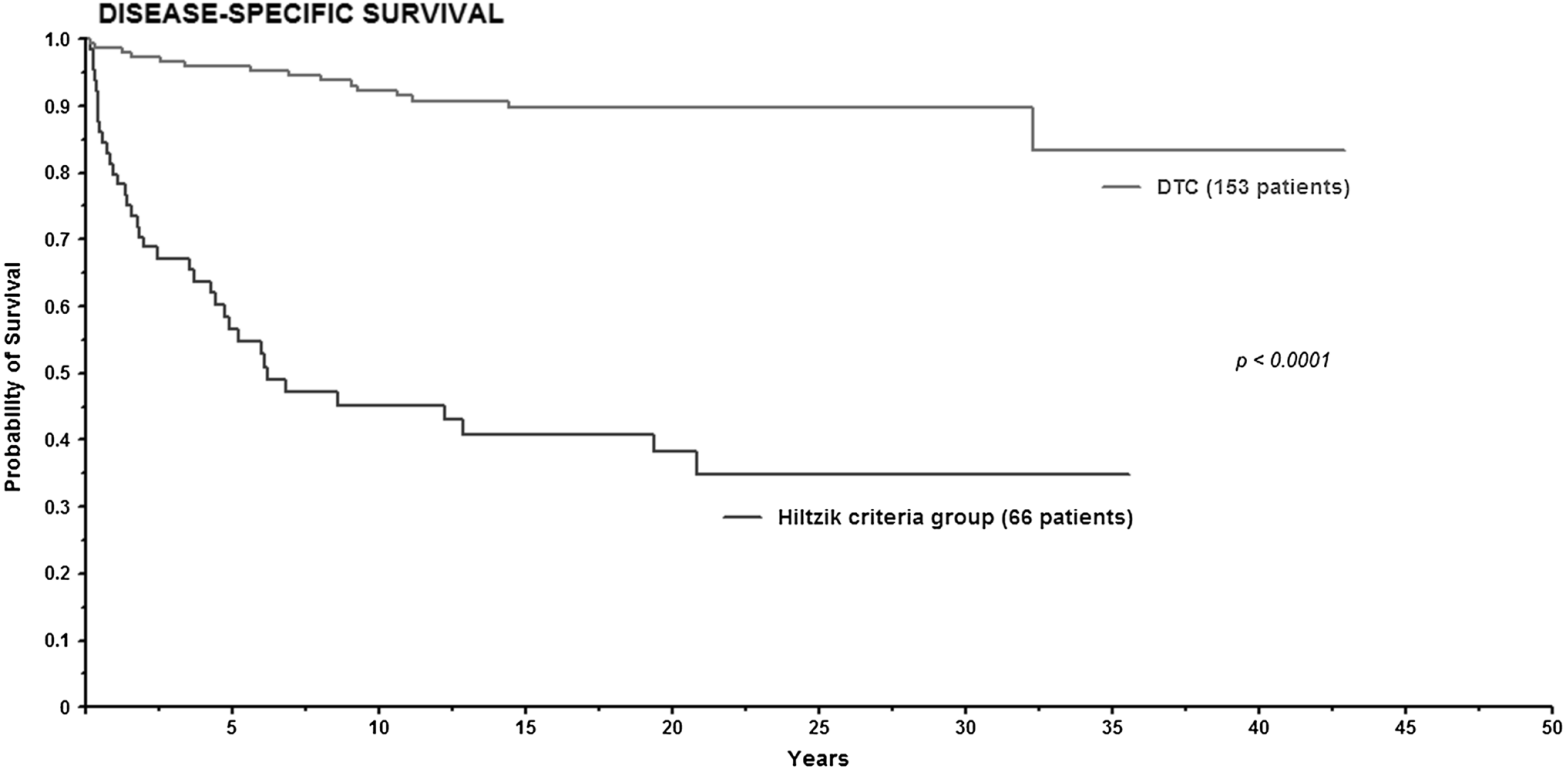
Kaplan–Meier curve for disease specific survival in differentiated thyroid carcinoma and Hiltzik criteria group

Figure 4 shows Kaplan–Meier survival curves for DTC and Hiltzik criteria group for DSS. In the DTC group the 5-, 10- and 20-year survival was 95, 92 and 90%, respectively. In the Hiltzik criteria group the 5-, 10- and 20-year survival was 71, 55 and 48%, respectively. Log rank test showed significantly difference between the groups (*P* < 0.0001).

## Discussion

This study is the first to evaluate the prognostic significance of the Hiltzik criteria (tumor necrosis and mitosis) on NAFCTC long time survival. In general DTC displays an excellent prognosis and since recurrence and death from disease can occur after decades, a long follow-up time is important. In the present study, all patients were followed for at least 20 years. This provides an accurate 20-year survival analysis with no censoring due to a short follow-up time, which strengthens the study. Limitations are the treatment at a single institution, a relatively small number of patients and variation in treatment strategy since at the time no national or regional treatment guidelines were available for standardization.

An initial analysis of the study group was performed in 1998 [12]. It was based on a relatively short follow-up time with a median of 10.2 years. Age above 45 years, distant metastasis, tumor necrosis, extrathyroidal invasion and p53-expression were found to be significant prognostic indicators. At that time PDTC was not yet recognized as a separate histological entity, and consequently some of the specimens would be re-classified as PDTC today explaining the relatively poor survival results initially presented.

The aim of the present study was to evaluate long-term prognostic factors with focus on tumor necrosis and mitosis. This would allow differentiation in survival analysis between DTC and PDTC according to the Hiltzik criteria [7], which Gnemmi el al. [11] found to be similar to TURIN criteria in predicting prognosis. Our study based on very long observation time confirms the results by Gnemmi et al.

Age is generally known to be one of the most influential prognostic factors in DTC, but there is still disagreement on where to set the cut-point. In numerous studies different cut-points are suggested such as 40, 45, 50 and 60 years [5, 15–20]. In our study 45 years was the cut-point, which is in accordance to the UICC/TNM staging system for DTC [14]. In the multivariate analysis of our series age is significant in OS but not in DSS.

Distant metastasis is also a very strong prognostic factor in DTC. This has been shown in several others studies [16, 18, 21] and is yet reconfirmed. Because of standardization of radioiodine therapy prognosis may have slightly improved for patients with distant metastasis, especially in younger patients with lung metastases and ^131^I-uptake [22].

Godballe et al. [12] identified both tumor size and extrathyroidal invasion as significant prognostic factors in univariate analysis, but the significance of size disappeared in the multivariate analysis. In the present long-term study tumor size remained significant in multivariate analysis of NAFCTC, suggesting that both tumor size and extrathyroidal invasion contain prognostic information.

In univariate analysis histological type (papillary versus follicular) was significant in OS and DSS. Patients with follicular carcinoma had a considerable less favorable prognosis than patients with papillary carcinoma. However, this significance was not found in multivariate analysis. The result is confirmed in some studies [5, 16, 17], while other studies find a prognostic difference [19–21].

Tumor necrosis and tumor mitosis were significant independent prognostic factors in OS and DSS in multivariate analysis. As mentioned above these factors are used in histology grading for PDTC, which is a relatively new diagnostic entity [9]. According to the TURIN proposal information regarding convoluted nuclei and growth pattern (trabecular/insular/solid) is also required to classify PDTC. In our study information about growth pattern was not evaluated. Thus application of the TURIN proposal was not possible. However, the Hiltzik criteria only required the high-grade features tumor necrosis and/or mitosis. When the NAFCTC patients were divided according to the Hiltzik criteria (Fig. 2), 153 patients were classified as DTC and 66 patients as PDTC. Compared to several other studies this model displayed similar 10-year survival exceeding 90% [5, 19, 23] for DTC. In PDTC 5-year OS has been reported from 60 to 73% [7, 11, 24], which is comparable to the 5-year OS at 57% in this study. Our findings support that tumor necrosis and/or mitosis according to Hiltzik criteria identify a group of patients with an intermediate prognosis equivalent to PDTC.

Due to the long follow-up time this study showed some interesting recurrences. Four patients in our study had their first recurrence more than 10 years after initial treatment. In one case the patient had the first recurrence 27 years after initial treatment and died 5 years later from thyroid carcinoma with distant metastases in multiple organs. This has also been observed in other long-term follow-up studies [25]. The findings emphasize the importance of a long follow-up time.

## Conclusion

Tumor necrosis and mitosis are highly significant prognostic indicators in analysis of long time survival of nonanaplastic follicular cell-derived thyroid carcinoma indicating that a simplification of the actually used criteria for poorly differentiated carcinomas may be justified.
